# Peripheral CB1 Receptor Neutral Antagonist, AM6545, Ameliorates Hypometabolic Obesity and Improves Adipokine Secretion in Monosodium Glutamate Induced Obese Mice

**DOI:** 10.3389/fphar.2018.00156

**Published:** 2018-03-20

**Authors:** Haiming Ma, Guina Zhang, Chunrong Mou, Xiujuan Fu, Yadan Chen

**Affiliations:** ^1^Department of Pharmacy, China-Japan Union Hospital of Jilin University, Changchun, China; ^2^Linyi City 120 Emergency Command Center, Linyi, China; ^3^People’s Hospital of Rizhao, Rizhao, China; ^4^Department of Pharmacy, The Second Hospital of Jilin University, Changchun, China

**Keywords:** CB1 receptor, AM6545, peripheral, obesity, antagonist

## Abstract

Effect of peripheral cannabinoid receptor 1 (CB1R) blockade by AM6545 in the monosodium glutamate (MSG)-induced hypometabolic and hypothalamic obesity was observed, and the impact on intraperitoneal adipose tissue and adipokines was investigated. The MSG mice is characterized by excessive abdominal obesity, and combined with dyslipidemia and insulin resistance. 3-Week AM6545 treatment dose-dependently decreased the body weight, intraperitoneal fat mass, and rectified the accompanied dyslipidemia include elevated serum triglyceride, total cholesterol, free fatty acids, and lowered LDLc level. Glucose intolerance and hyperinsulinemia were also alleviated. But AM6545 didn’t affect the food-intake consistently through the experiment. In line with the reduction on fat mass, the size of adipocyte was reduced markedly. Most interestingly, AM6545 showed significant improvement on levels of circulating adipokines including lowering leptin, asprosin and TNFα, and increasing HMW adiponectin. Correspondingly, dysregulated gene expression of lipogenesis, lipolysis, and adipokines in the adipose tissue were nearly recovered to normal level after AM6545 treatment. Additionally, western blot analysis revealed that AM6545 corrected the elevated CB1R and PPARγ protein expression, while increased the key energy uncoupling protein UCP1 expression in adipose tissue. Taken together, the current study indicates that AM6545 induced a comprehensive metabolic improvement in the MSG mice including counteracting the hypometabolic and hypothalamic obesity, and improving the accompanied dyslipidemia and insulin resistance. One key underlying mechanism is related to ameliorate on the metabolic deregulation of adipose tissue, the synthesis and secretion of adipokines were thus rectified, and finally the catabolism was increased and the anabolism was reduced in intraperitoneal adipose tissue. Findings from this study will provide the valuable information about peripheral CB1R antagonist in managing hypometabolic obesity.

## Introduction

Obesity and closely related cluster of metabolomic diseases such as hyperlipidemia and diabetes, are becoming pandemic chronic disease worldwide, and seriously affect the quality of human life and also constitute a heavy burden for the health system of all countries (Cao, 2014). Innovative therapeutic interventions to alleviate these metabolic disorders are in urgent demand. Since, the discovery of the cannabinoid receptor 1 (CB1R) system, and the subsequent disclosure of its critical roles in the developing and progression of obesity, the pursuit for effective CB1R antagonists to manage morbid obesity haven’t lost its momentum (Sharma et al., 2014; Murineddu et al., 2017). After the well-known first in-class selective CB1R antagonist/inverse agonist SR141716 (rimonabant) approved by the European Medicines Agency (EMA) and then withdrawn from market in 2008, the second generation of CB1R antagonists are becoming the strategy in progress to solve the problems derived from the first generation of CB1R antagonists such as SR141716 (Wu et al., 2011; Chorvat, 2013). Which include the developing of peripherally biased neutral antagonists and inverse agonists with limited central nervous system (CNS) permeability (Janero, 2012). Several excellent lead compounds have been described in succession over the past few years, examples are the non-brain-penetrant neutral CB1R antagonist AM6545 and TXX-522, and the peripheral inverse agonists TM38837 and JD-5037 (Cluny et al., 2010; Chorvat et al., 2012; Klumpers et al., 2013; Chen et al., 2017). Their anti-obesity effect and pharmacological mechanism of action are frequently evaluated in diet-induced obesity (DIO) or genetic obese rodent models (Ravinet Trillou et al., 2003; Jourdan et al., 2010; Chen et al., 2017). However, to some extent, it’s difficult to accurately illuminate the peripheral mechanisms of action of the peripheral CB1R antagonists with these animal models, because it is hard to thoroughly preclude the impact of CNS, especially hypothalamus-associated roles in its effect.

Monosodium glutamate (MSG) rats or mice, a hypometabolic and hypothalamic obese rodent model made by injecting with MSG shortly after birth, might be a feasible model to investigate the efficacy and mechanism of peripheral CB1R antagonists, because most of the hypothalamic arcuate nucleus (ARC) and the adjacent ventromedial nucleus (VMN) are damaged by MSG (Zhang et al., 2010; Hernández-Bautista et al., 2014). This model had recently been used to address the peripheral anti-obesity effect of SR141716 after chronic treatment (Chen et al., 2013). In contrast to the DIO model, neuroendocrine and metabolic dysfunctions are typical phenotypes observed in the MSG rats or mice (Hernández-Bautista et al., 2014). They are not hyperphagic but hypometabolic, and display classic abdominal obesity, and also accompanied with several other metabolic dysfunctions including hyperinsulinemia, hyperlipidemia, and hyperleptinemia (Chen et al., 2008; Hernández-Bautista et al., 2014).

White adipose tissue (WAT) accumulated excess triacylglycerol as fat under conditions of morbid obesity, its volume expanded extensively, whereas its energy expenditure is reduced (Smith and Kahn, 2016). Meanwhile, the expression and secretion of adipose derived hormones (referred to as adipokines or adipocytokines), include adiponectin, leptin, asprosin, tumor necrosis factor-α (TNF-α), resistin, visfatin, etc. are dysregulated (Cao, 2014; Ouchi, 2016). This has been recognized as a key etiological factor of obesity-induced disorders, because adipokines mediate the crucial crosstalk between adipose tissue and other key metabolic tissues, especially the liver, muscle, and pancreas, as well as the brain (Kuryszko et al., 2016; Smith and Kahn, 2016). The improvement on the levels of local and systemic adipokines may greatly contribute to the alleviation of obesity and related metabolomic disorders. The CB1R is expressed at high levels in the adipose tissues among various peripheral tissues (Despres et al., 2006). The mechanism of action of SR141716 in decreasing visceral fat mass and improving insulin insensitivity are presumed to be related to the increase of adiponectin, and the reduction of TNF-α and leptin (Mohapatra et al., 2009; Ge et al., 2013). However, the impact of a pure peripheral CB1R antagonist on adipokines in management of obesity has not yet been well-clarified.

In current study, AM6545, a peripheral CB1R targeted neutral antagonist was used as a probe compound to investigate the efficacy of peripheral CB1R antagonism on the hypometabolic and hypothalamic obesity of MSG mice. Meanwhile the roles of adipose tissue, particularly the synthesis and secretion of crucial adipokines that closely correlated with metabolic homeostasis were studied in the experiment. To this aim, body weight gain, food intake, systemic adipokines as well as adipose histopathology were examined. In addition, the mRNA encoding genes of adipokines and the genes involve in lipids metabolism in adipose tissue were also observed.

## Materials and Methods

### Animal and Experimental Protocols

Pregnant ICR mice were obtained from the experimental animal center of Beijing Medical Science Academy (Beijing, China) and maintained in an air-conditioned room under controlled illumination (12-h light/dark cycle), temperature (23 ± 1°C) and humidity of 40–60%, had free access to standard rodent chow and water throughout the experiment. Neonatal ICR mice were injected subcutaneously with 4 mg/g body weight MSG (Sigma–Aldrich, St. Louis, MO, United States) (MSG mice) for 8 consecutive days after birth to induce obesity. Saline water injected mice were used as normal control (NC). After weaning, mice were kept for another 5 months under normal condition. Then, the male MSG mice were divided into three different groups, i.e., model control (MC) and the two AM6545 (3 and 10 mg/kg body weight) treated MSG mice group (*n* = 8), based on their initial body weight. Either vehicle (5% Tween 80 and 5% dimethyl Sulfoxide in sterile salin) or AM6545 (Sigma–Aldrich, St. Louis, MO, United States) was administered by IP injection daily for 3 weeks. The dosage of AM6545 was chosen to treat animals as previously described. Body weight and food intake were recorded every day during the treatment period. On the last day of the experiment, overnight fasted mice were sacrificed by decapitation. Plasma was collected for immediate assessment of serum biochemical parameters. The liver and intraperitoneal adipose tissues were excised and stored at -80°C for subsequent RNA extraction and quantitative RT-PCR analysis.

All animal handling and experiments were performed strictly in accordance with the recommendations of the Guide for the Care and Use of Laboratory Animals of the National Institutes of Health. The experimental protocol was approved by the Animal Experimental Ethics Committee of the Jilin University.

### Oral Glucose Tolerance Test (OGTT)

After 2 weeks of treatment, mice were fasted for 6 h before the test. OGGT was performed by gavage a glucose bolus (2 g/kg), and blood glucose levels were determined at 0, 30, 60, 90, and 120 min by using the glucometer (Johnson & Johnson, United States) through the tail tip. The area under the glucose curve (AUC) generated from blood glucose recordings were calculated.

### Biochemical Analysis and Serum Adipokine Detection

Serum levels of total cholesterol (TC), triglycerides (TG), and free fatty acids (FFAs) levels were determined using enzymatic colorimetric methods with commercial kits according to the manufacturer’s instructions (Rongsheng Biotech, Shanghai, China).

Serum insulin, leptin, TNFα, and HMW adiponectin concentrations were assayed by using MILL IPLEX MAP Mouse Metabolic Magnetic Bead Panel kit (Millipore, Billerica, MA, United States) with FlexMAP3D. Serum asprosin was determined by ELISA (abbexa, Cambridge, United Kingdom).

### Histopathological Examination

Samples of intraperitoneal adipose tissue were resected and fixed with 10% formaldehyde phosphate buffered saline (PBS, pH = 7.4) and then embedded in paraffin, sectioned, stained with hematoxylin/eosin, and analyzed by microscopy and morphometry with an Olympus BX51TF microscope (Olympus, Co., Tokyo, Japan).

### qPCR Analysis

Total RNA from the intraperitoneal adipose tissue of the mice was prepared with the Trizol RNA preparation kit following the manufacturer’s recommended procedures (Gibco-BRL, Grand Island, NY, United States) and converted to cDNA with oligo dT primers by using a cDNA synthesis kit (Takara Biotechnology, Co. Ltd., Dalian, China) in a thermocycler (Mastercycler, Eppendorf, Hamburg, Germany). QPCR with the ABI PRISM 7500 Sequence Detection System (Applied Biosystems, Foster City, CA, United States) was performed using the ABI Power SYBR Green PCR Master Mix (Applied Biosystems, Warrington, United Kingdom). The primer sequences used are listed in Supplementary Table 1. All reactions were carried out in triplicate. mRNA expression of the genes was normalized to that of β-actin.

### Western Blot Analysis

Adipose tissues were homogenized in ice-cold RIPA buffer (1 mM EDTA, 1% Triton X-100, 0.1% SDS, 1 mM Na_3_VO_4_, 1 mM PMSF, 10 mM pyrophosphate, 100 mM NaF, and 1 mg/ml bacitracin). Tissue lysates were centrifuged at 12,000 *g* for 15 min at 4°C and protein concentrations in the supernatant were determined with bicinchoninic acid assay. Proteins were resolved by 10% SDSPAGE, transferred to PVDF membrane and then blocked with 5% milk blocking buffer (Tris-buffered saline with 0.1% Tween 20). The membranes were, respectively, probed with rabbit polyclonal antibody against CB1R and PPARγ, rabbit monoclonal antibody against UCP1 (Abcam, Cambridge, MA, United States), and rabbit polyclonal antibody against β-actin (Santa Cruz Biotechnology, Inc., Santa Cruz, CA, United States) overnight at 4°C, followed by horseradish peroxidase-conjugated secondary antibody (Santa Cruz Biotechnology, Inc., Santa Cruz, CA, United States). Detection of immunoreactive band was achieved using enhanced chemiluminescence detection reagents (Applygen Technologies, Inc., Beijing, China), and the intensity of the corresponding bands were analyzed with a ChemiImager 5500 system (Alpha Innotech, San Leandro, CA, United States). The expression of proteins was normalized to that of β-actin.

### Statistical Analysis

All values are expressed as mean ± SEM. Statistical analyses were assessed by one-way ANOVA followed by the Tukey’s multiple comparison tests with SPSS (SPSS, Inc., Chicago, IL, United States) to compare the experimental groups. *p* < 0.05 was considered statistically significant.

## Results

### Effects of AM6545 on Metabolic Parameters in MSG Mice

**FIGURE 1:**
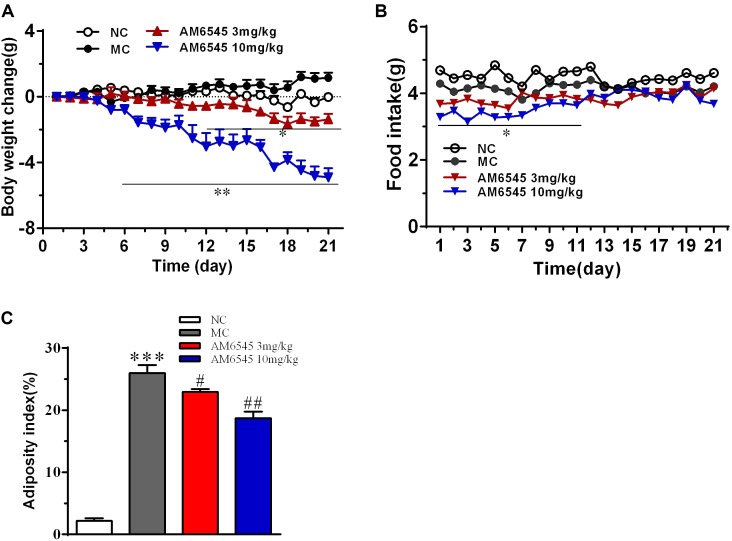
Effect of AM6545 on **(A)** net body weight change, **(B)** food intake, and **(C)** intraperitoneal adipose weight index in MSG mice. Net body weight change during administration period was calculated for individual mice and then averaged. NC, normal control; MC, model control; values are mean ± SEM, *n* = 8 per group; ^∗^*p* < 0.05, ^∗∗^*p* < 0.01, ^∗∗∗^*p* < 0.001 versus NC group; #*p* < 0.05, ##*p* < 0.01 versus MC group.

**FIGURE 2:**
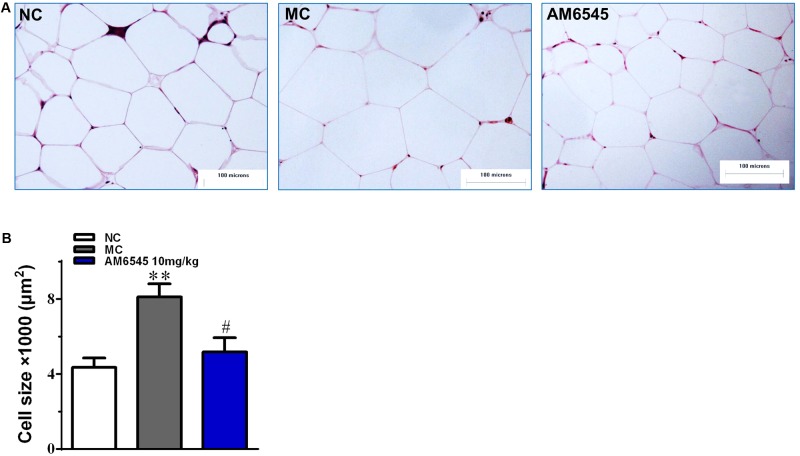
The impact of AM6545 on intraperitoneal adipose tissue mass and size in the MSG mice. **(A)** Representative photomicrograph of intraperitoneal adipose section. **(B)** The average size of adipocytes in the intraperitoneal adipose tissue. The bar indicates 100 μm in the panels (HE: 40×). NC, normal control; MC, model control; values are mean adipocyte size ± SEM, *n* = 8 per group; data were analyzed by one-way ANOVA followed by the multiple comparison tests. ^∗∗^*p* < 0.01 versus NC group; #*p* < 0.05 versus MC group.

Compared to the NC mice, the 6-month old MSG mice are stunted and markedly obese with excessive intraperitoneal adipose tissue and significantly increased body weight (41.5 ± 2.8 vs. 27.3 ± 1.4 g, respectively, at the beginning of the study, *p* < 0.01). Compared with the vehicle treated mice, 3-week treatment with AM6545 dose dependently reduced the body weight; significant decrease was observed from 6 to 13 days in 10 mg/kg group (*p* < 0.01) and 3 mg/kg group (*p* < 0.05), respectively (**Figure 1A**). The weigh-loss effect lasted till the end of the study. 3 mg/kg AM6545 showed no impact on food intake of MSG mice throughout the test, whereas 10 mg/kg produced a transient reduction in the initial period of administration, then it rebounded to the equivalent amount of MC’s (**Figure 1B**). Intraperitoneal adipose tissue was decreased 11.7 and 35.3% by 3 and 10 mg/kg AM6545 respectively at the end of the treatment (**Figure 1C**). In parallel with this, histopathological analysis of the adipose tissue revealed the size of adipocytes was significantly diminished by AM6545 (*p* < 0.05 for the comparison of AM6545 vs. MC) (**Figure 2**).

### Effects of AM6545 on Glucose Tolerance

**FIGURE 3:**
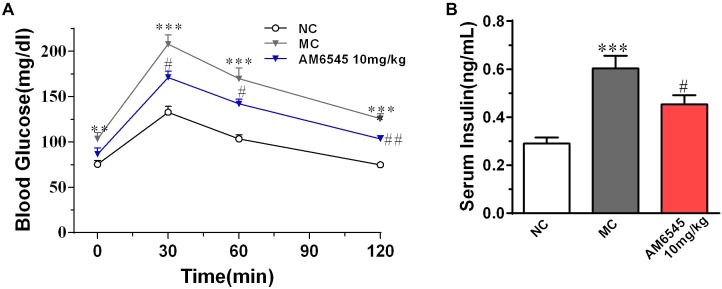
Effect of AM6545 on glucose tolerance and serum insulin level in MSG mice. **(A)** Oral glucose tolerance tests (OGTT) were performed after 2 weeks treatment. Mice were fasted for 6 h and then subjected to OGTT, blood glucose was tested at the indicated time. **(B)** Serum insulin level. NC, normal control; MC, model control; values are mean ± SEM, *n* = 8 per group; *^∗∗^p* < 0.01, *^∗∗∗^p* < 0.001 versus NC group; *#p* < 0.05, *##p* < 0.01 versus MC group.

The MSG obese mice also characterized with apparent glucose intolerance, which was indicated by the markedly increased blood glucose under glucose load (**Figure 3A**) and significantly increased hyperinsulinemia (**Figure 3B**). Compared to the vehicle treated MSG mice, AM6545 treatment produced markedly lower blood glucose levels at 30, 60, and 120 min after glucose loading (**Figure 3A**). Additionally, AM6545 also significantly lowered the fasting insulin level (*p* < 0.05) (**Figure 3A**). These results indicate subchronic administration with AM6545 could alleviate the insulin resistance in the MSG mice.

### Effects of AM6545 on Serum Lipid Profiles and Adipokines

**FIGURE 4:**
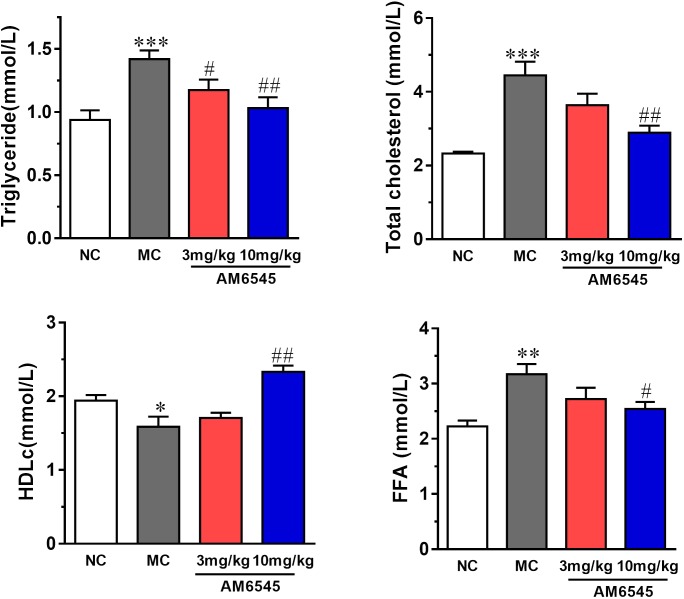
Effect of AM6545 on serum lipids in MSG mice. NC, normal control; MC, model control; values are mean ± SEM, *n* = 8 per group; *^∗^p* < 0.05, *^∗∗^p* < 0.01, *^∗∗∗^p* < 0.001 versus NC group; *#p* < 0.05, *##p* < 0.01 versus MC group.

Compared to NC group, the MSG mice displayed apparent dyslipidemia, including hypertriglyceridemia, increased total cholesterol, FFA, and decreased HDLc (**Figure 4**). After 3-weeks treatment, serum TG was lowered by 17.3 and 27.4% by 3 mg/kg (*p* < 0.05) and 10 mg/kg, respectively (*p* < 0.01). However, the serum FFA and TC were only notably reduced by the higher dose of AM6545 (**Figure 4**). Meanwhile, serum HDLc was also found increased by 10 mg/kg AM6545 after the chronic treatment.

**FIGURE 5:**
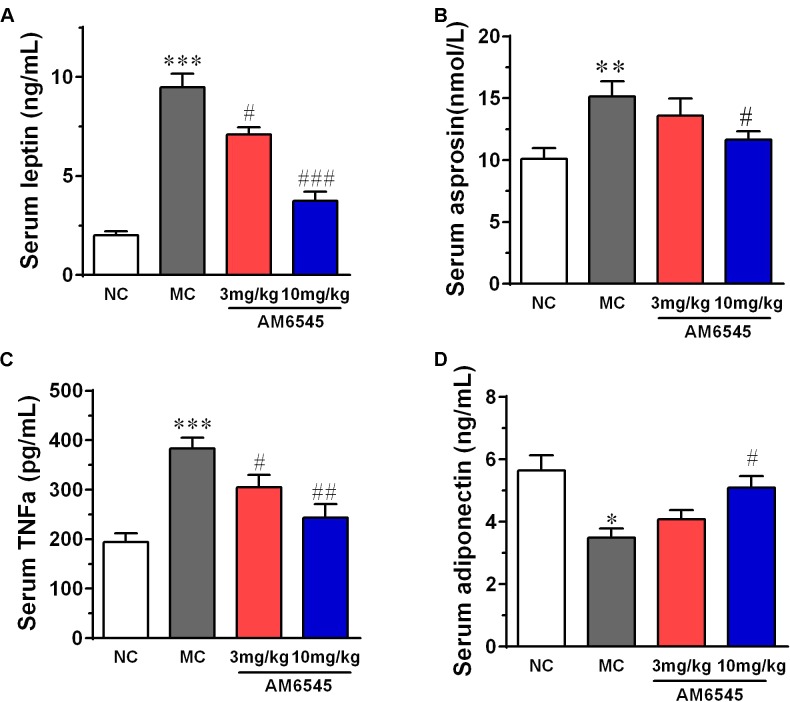
Effect of AM6545 on serum adipokines in MSG mice. **(A)** Serum leptin. **(B)** Serum asprosin. **(C)** Serum TNF. **(D)** Serum adiponectin. NC, normal control; MC, model control; values are mean ± SEM, *n* = 8 per group; *^∗^p* < 0.05, *^∗∗^p* < 0.01, *^∗∗∗^p* < 0.001 versus NC group; *#p* < 0.05, *##p* < 0.01, *###p* < 0.001 versus MC group.

When compared with that of NC mice, MSG mice displayed apparent deregulated circulating adipokines. Serum leptin concentration in MSG mice was increased by sixfold (**Figure 5A**), which is in consistent with previous report in the MSG-induced rodent model. Meanwhile, asprosin, a protein hormone that assumed to originate from adipose tissue, and TNFα were also marked elevated by about twofold (**Figure 5B**). In contrast, the circulating HMW adiponectin was significantly reduced (**Figure 5D**). However, 3 week treatment with AM6545 effectively corrected the hyperleptinemia, decreased the TNFα content, and increased the HMW adiponectin in a dose dependent manner, with 10 mg/kg displayed more apparent improvement. Moreover, the higher dose of AM6545 also strikingly decreased serum asprosin content (**Figure 5**).

### Protein and Gene Expression Analysis in Adipose Tissue

**FIGURE 6:**
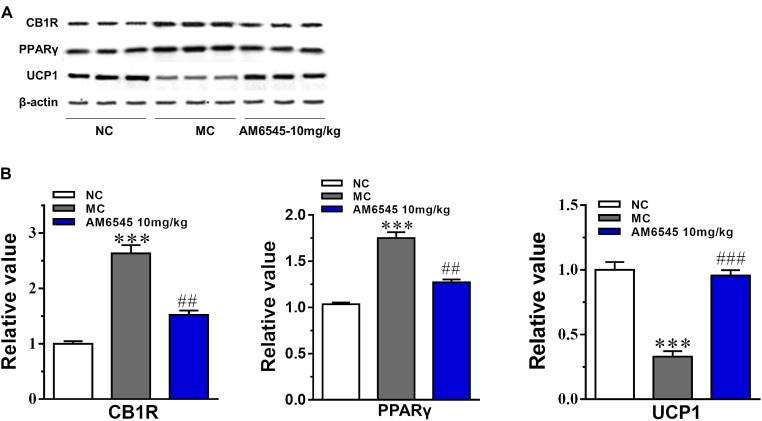
Effect of AM6545 on protein expression in intraperitoneal adipose tissue of the MSG mice. **(A)** The protein expression of CB1R, PPARγ, and UCP1 analyzed by Western blot assay. **(B)** Quantification of the results in **(A)**. Results are normalized to β-actin in the correspondent group, and expressed as relative expression compared with that in NC group. NC, normal control; MC, model control; values are mean ± SEM, *n* = 3; *^∗∗∗^p* < 0.001 versus NC group; *##p* < 0.01, *###p* < 0.001 versus MC group.

The expression of CB1R and concentration of endocannabinoids are usually increased under condition of obesity. To uncover the mechanism of AM6545 in regulating lipids metabolism in adipose tissue, we detected the protein expression of CB1R, PPARγ, and UCP1 in intraperitoneal adipose tissue. As shown in **Figure 6**, the CB1R expression was up-regulated by nearly two times in adipose tissue in MSG mice relative to that of the NC mice (*p* < 0.001), indicating the existence of ECS (endocannabinoid system) overactivity. In line with this, the protein expression of PPARγ, the nuclear transcriptional regulator that plays key roles in adipocyte differentiation and adipose formation, was also elevated strikingly (*p* < 0.001). Whereas, the major thermogenesis protein, uncoupling protein 1 (UCP1) was down-regulated in the adipose tissue (*p* < 0.001). 3-Weeks treatment with AM6545 reversed the elevated protein expression of CB1R and PPARγ, and up-regulated UCP1 content in adipose tissue of MSG mice (**Figure 6B**), signifying the over-activated ECS activity was effectively inhibited by peripheral CB1R antagonism, and the circumstance facilitating lipids and adipose accumulation was reverted by AM6545 interruption.

**FIGURE 7:**
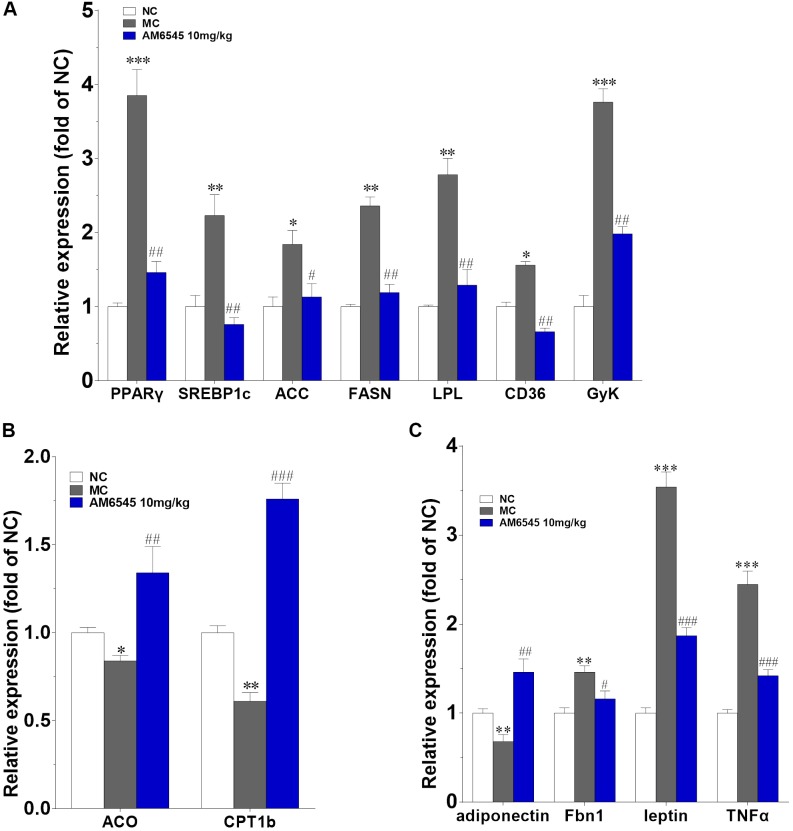
Effect of AM6545 on gene expression in intraperitoneal adipose tissue of the MSG mice. The gene expression of lipogenesis **(A)**, lipolysis **(B)**, and adipokines **(C)**. Results are normalized to β-actin and expressed as mean fold increase of mRNA ± SEM compared with NC group. NC, normal control; MC, model control; *n* = 3; *^∗^p* < 0.05, *^∗∗^p* < 0.01, *^∗∗∗^p* < 0.001 versus NC group; *#p* < 0.05, *##p* < 0.01, *###p* < 0.001 versus MC group.

Furthermore, the levels of mRNA encoding genes involved in fatty acid oxidation and energy expenditure, and crucial adipose-derived hormones related to systemic lipid metabolism and energy expenditure were also analyzed. In **Figure 7A**, the key lipogenic genes including acetyl CoA carboxylase (*ACC*), fatty acid synthase (*FAS*), *CD36* and lipoprotein lipase (*LPL*), and their upstream transcriptional regulator (*PPARγ* and *SREBP1c*) were all found markedly down-regulated by AM6545. Meanwhile, the key enzyme of fatty acids oxidation, the carnitine palmitoyltransferase Ib (*CPTIb*) and the acyl-coA oxidase (*ACO*), were increased (**Figure 7B**). Glycerokinase (GyK), the enzyme that is responsible for converting glycerol to alpha-glycerophosphate and is presumed to promote the esterification of free fatty acids to triglyceride, was also inhibited dramatically after treatment (**Figure 7A**). Furthermore, in corresponding with the circulating level, the gene expression levels of the adipokines leptin, TNFα and aspronin were found down-regulated, whereas adiponection was increased in the adipose after AM6545 treatment (**Figure 7C**).

## Discussion

In this study, we investigated the anti-obesity effects of AM6545, a peripheral restricted CB1R antagonist with poor brain permeability, on the hypometabolic and hypophagic obesity of MSG mice for the first time. This model is characterized by severe abdominal obesity, dyslipidemia and mild insulin intolerance symptoms, mimics the main features of human metabolic syndrome (Chen et al., 2008; Zhang et al., 2010; Sasaki et al., 2011). Different from the commonly used classical diet induced obese rodent model, hypothalamic centers that are responsible for centrally regulating food consumption and energy homeostasis will not play important roles in AM6545’s weight reduction in the MSG mice, and the effect of peripheral CB1R blockade could be observed more purely and apparently. Here, we noticed that subchronic administration of AM6545 could ameliorate the dyslipidemia, and induce significant body weight loss in a dose-dependent manner in the MSG mice, although no significant anorexia was observed throughout the experiment. Most importantly, the deregulated adipokines were found improved greatly at the end of the treatment. Furthermore, the catabolism of lipids was induced, whereas the anabolism of triglyceride was inhibited.

In contrast to previous report that AM6545 decreased feeding behavior in the high fat or high fat and sucrose induced obese mice (Cluny et al., 2010; Randall et al., 2010), AM6545 showed no consistent inhibition on the intake of stand rodent chow in the MSG mice, although a slight but non-significant decrease were displayed in the initial period of administration. This is in agreement with the report by Argueta and DiPatrizio (2017) that AM6545 also failed to modify the intake of standard chow diet in C57BL/6 mice. Meanwhile Boon et al. (2014) also didn’t find altered food intake in the 12-week high fat diet induced obese mice after 4-week AM6545 administration. On one hand, this discrepancy might be partially related to the different animal model, the high fat diet induced obese mice are hyperphagic, whereas the MSG mice are non-hyperphagic. However, the most important underlying reason may be associated with the distinct pathological mechanisms among different obesity models, the hypothalamus region of the MSG mice was damaged and thus the centrally CB1R antagonism will not effect, as mentioned above. Correspondingly, the basal food intake of the MSG mice was found significantly lower than that of the age matched littermates. Thus it can be concluded that the beneficial effects of subchronic AM6545 administration in the MSG mice are independent of the anorectic action.

Here, we found that the expression of CB1R in the adipose tissue of MSG mice was up-regulated robustly, which is similar to the finding on the MSG rat, and is also in agreement with previous report that components of the endocannabinoid system (i.e., cannabinoid receptors, and endocannabinoid biosynthetic and degradative enzymes) were dysregulated in both of the high fat diet induced and genetic obese rodent models (Jourdan et al., 2010; Chen et al., 2013). Meanwhile, in consistent with the increased CB1R expression, genes responsible for lipogenesis and triglyceride storage (*PPARγ* and *SREBP1c*, and their downstream target genes including *ACC*, *FAS,* and *LPL*) in the adipose tissue of MSG mice were also up-regulated markedly, and along with this, the expression of the lipolytic genes (*CPTIb* and *ACO*) were apparently down-regulated, signifying that the elevated adipose CB1R contributes to the lipogenesis and the storage of lipids within the tissue, and the inhibition of fatty acid β oxidation simultaneously. In line with this, the elevated protein expression of PPARγ and lowered protein expression of UCP1 also further confirmed this result. These alterations together disclosed why the MSG mice had excess fat mass under condition of reduced food consumption. However, AM6545 subchronic treatment recovered the abnormally increased expression of CB1R and PPARγ in adipose tissue to nearly normal levels. Meanwhile, the deregulated expression of lipogenic and lipolytic key enzymes were also restored, which leads to a normal or increased fatty acid catabolism and thermogenesis. Accordingly, the adipose tissue weight and size of adipocytes were lowered, and finally the body weight was reduced. The loss of intraperitoneal adipose tissue accounted for more than 50% of body weight reduction in both treatment groups. Additionally, accompanied with the improvement on adipose metabolism, the dyslipidemia was also rectified notably. To some extent, our current results of AM6545 are in good agreement with the effects of chronic SR141716 treatment for 6 weeks on body weight and dyslipidemia in MSG rats and diet-induced obese mice, when the impact of SR141716 on food intake was already gone, and the anti-obesity effect was totally attributed to the peripheral CB1R antagonism (Ravinet Trillou et al., 2003; Chen et al., 2013).

As an important endocrine organ and a sensor/modulator of energy homeostasis, the metabolism deregulation of adipose tissues play critical roles in the developing and progression of obesity (Gandhi et al., 2010; Cao, 2014). Adipokines secreted by adipose tissue directly and/or indirectly affect nearby or remote tissues through endocrine, paracrine, autocrine or juxtacrine, modes of action, and their dysfunction has been linked to a wide range of metabolic disorders (Gandhi et al., 2010; Kuryszko et al., 2016; Ouchi, 2016). Here we found circulating leptin and aspronin were remarkably increased, whereas HMW adiponectin was decreased in the MSG mice. Which are in consistent with previous report in the MSG and diet induced obese mice or rat, indicate the obesity of the MSG mice are closely related with the dysregulated secretion of adipokines. AM6545 subchronic treatment could rectify these abnormal production and secretion of adipokines from adipose tissue. Correspondingly, we found strong up-regulation of *adiponectin* and down-regulation of *TNFα*, *leptin* and *Fbn1* (the encoding gene of apronin) mRNA in WAT. HMW adiponectin is the active style of adiponectin, the lowered levels of it is associated with the etiopathology of obesity and diabetes (Ouchi, 2016). Thus whose increase contributes to the improvement of metabolism elicited by AM6545. Asprosin, the C-terminal cleavage product of profibrillin, is a recently identified white adipose derived adipokine and has been confirmed to be capable to regulate systemic glucose homeostasis through inducing hepatic glucose output (Greenhill, 2016; Zhang et al., 2017). Asprosin is pathologically elevated under condition of insulin resistance and obesity, and its reduction could contribute to the amelioration of these abnormalities (Romere et al., 2016). Leptin possesses pivotal roles in regulating appetite and adiposity, and also affects the synthesis and secretion of HMW adiponectin (Cao, 2014; Ouchi, 2016). Leptin resistance had been confirmed to participate in the pathogenesis of obesity, the related oxidative stress and inflammation (Cao, 2014). Amelioration of hyperleptinemia and leptin resistance had been demonstrated to be one key underlying mechanism of CB1R antagonism in alleviation of obesity. Additionally, elevation of TNFα, a proinflammatory cytokine, has been widely accepted as an important mechanistic connection between obesity and its complications (Tzanavari et al., 2010). In current experiment, we found serum asprosin and leptin level were almost recovered to normal level after treatment with AM6545. This may be originated from the improvement in the metabolism of adipose tissue by AM6545, and conversely these adipokines may further contribute to the relievement of obesity and insulin resistance in MSG mice. Indeed, the glucose intolerance and hyperlipidemia were strikingly improved by subchronically treatment with AM6545. This result is in agreement with that in the high fat diet-induced obesity and pre-diabetes mice (Cluny et al., 2010; Argueta and DiPatrizio, 2017).

## Conclusion

By improving the deregulated metabolism of adipose tissues through specific peripheral CB1R antagonism, we found the dysregulated synthesis and secretion of adipokines were thus rectified, and furthermore a comprehensive metabolic improvement including counteracting obesity, improving dyslipidemia and insulin resistance was realized by AM6545 in the MSG mice. Findings from this study will provide the valuable information about peripheral CB1R antagonist in managing hypometabolic obesity.

## Author Contributions

Conceived and designed the experiments: GZ, YC, and HM. Performed the experiments: GZ, CM, HM, and YC. Analyzed the data: GZ, YC, XF, and HM. Wrote the paper: GZ, YC, XF, and HM.

## Conflict of Interest Statement

The authors declare that the research was conducted in the absence of any commercial or financial relationships that could be construed as a potential conflict of interest.
