# Supplementary material for: Peripheral CB1 Receptor Neutral Antagonist, AM6545, Ameliorates Hypometabolic Obesity and Improves Adipokine Secretion in Monosodium Glutamate Induced Obese Mice
Source: Front Pharmacol. 2018 Mar 20;9:156. doi: 10.3389/fphar.2018.00156 (PMC5869198; doi:10.3389/fphar.2018.00156)
Supplement: Supplementary file 1 [file Table_1.DOCX]

Table 1. The primer sequences used for qPCR.

| **Genes** | **Forward primers** | **Reverse primers** |
| --- | --- | --- |
| PPAR γ | 5'-CAGCAACCATTGGGTCAGCTC-3' | 5'-CCAGAGCATGGTGCCTTCGCT-3' |
| SREBP1c | 5'- AAGACAGATGCAGGAGCCAC-3' | 5'- ATGGTCCCTCCACTCACCAG-3' |
| ACC | 5'-CACCTGATGACACCTGTGCT-3' | 5'-CAGCACCGAGACTGAACTGT-3' |
| FASN | 5'-GCTGGCATTCGTGATGGAGTCGT-3' | 5'-AGGCCACCAGTGATGATGTAAC-3' |
| CD36 | 5'-AAGCTATTGCGACATGATT-3' | 5'-GATCCGAACACAGCGTAGAT-3' |
| LPL | 5'- CCTACAAAGTGTTCCATTACCA -3' | 5'- CTCGCTCTCGGCCACTGT -3' |
| GyK | 5'-TTCCAGGAAATAATAACTTTGTC-3' | 5'-CACTGCACTGAAATACGTGCT-3' |
| ACO | 5'- TTAAACACCCACCCACCAAG-3' | 5'- CGAAAGCCTGGAGGTAAAGA-3' |
| CPT1b | 5'-TGCCTTTACATCGTCTCCAA-3' | 5'-GGCTCCAGGGTTCAGAAAGT-3' |
| Adiponectin | 5'-TGTTGGAATGACAGGAGCTGA -3' | 5'- CACACTGAACGCTGAGCGATAC-3' |
| Leptin | 5'- CAGCTGCAAGGTGCAAGAAG-3' | 5'- GATACCGACTGCGTGTGTGA-3' |
| Fbn1 | 5'-GTCCGAGCCGCTAGTCCA-3' | 5'-ACTGTCCGGCTGTCCTGATG-3' |
| TNF-α | 5'-TGGTGCCTGGTCTGATGATG-3' | 5'-GTGGTAACCGCTCAGGTG-3' |
| β-actin | 5'-TAAAGACCTCTATGCCAACACAG-3' | 5'-CACGATGGAGGGGCCGGACTC-3' |
